# Fundamentals and Applications of the Receiver Operating Characteristic Curve Analysis in the Research of Endothelial Dysfunction in Chronic Kidney Disease

**DOI:** 10.3390/life12091402

**Published:** 2022-09-09

**Authors:** Stefanos Roumeliotis, Samar Abd ElHafeez, Graziella D’Arrigo, Maria Fusaro, Daniela Leonardis, Sabrina Mezzatesta, Giovanni Tripepi

**Affiliations:** 1 Division of Nephrology and Hypertension, 1st Department of Internal Medicine, AHEPA Hospital, School of Medicine, Aristotle University of Thessaloniki, 54636 Thessaloniki, Greece; 2 Epidemiology Department, High Institute of Public Health, Alexandria University, Alexandria 21561, Egypt; 3 Clinical Epidemiology of Renal Diseases and Hypertension Unit, Institute of Clinical Physiology, 89124 Reggio Calabria, Italy; 4 National Research Council (CNR), Institute of Clinical Physiology (IFC), 56124 Pisa, Italy; 5 Department of Medicine, University of Padua, 35129 Padua, Italy

**Keywords:** diagnostic test, discriminatory power, classification, endothelial dysfunction, receiver operating characteristic curves, sensitivity, specificity, statistical test

## Abstract

Endothelial dysfunction (ED) starts early in chronic kidney disease (CKD) and is the hallmark of atherosclerosis in these patients. During recent years, numerous markers have emerged, aiming to predict the onset of ED in CKD patients. Therefore, there is a need to evaluate and assess the discriminatory ability (or diagnostic accuracy) of such a marker (i.e., the ability to correctly classify individuals as having a given disease or not) and identify the optimal cut-off value. A receiver operating characteristic (ROC) curve analysis has been used in the majority of the research papers evaluating the predictive ability of a marker of ED. It is a graphical plot combining pairs of sensitivity (true positive rate) on the *y* axis and the complement of specificity (1—specificity, false positive rate) in the *x* axis, corresponding to several of the cut-off values covering the complete range of possible values that this test/marker might take. Herein, using a series of practical examples derived from clinical studies on ED in the special population of CKD, we address the principles, fundamentals, advantages and limitations regarding the interpretation of the ROC analysis.

## 1. Introduction

Chronic kidney disease (CKD) is a worldwide public health problem with increased prevalence and incidence. CKD and especially end stage kidney disease (ESKD) patients have an increased risk for cardiovascular (CV) mortality and morbidity, which is partially attributed to the development of atherosclerosis and arterial stiffness which is very pronounced in these patients [1]. Endothelial dysfunction (ED), the hallmark of atherosclerosis, is present even at early CKD stages and its prevalence progressively increases in parallel with the progression of CKD to end stage kidney disease (ESKD) [2]. Among others, inflammation and oxidative stress (OS) are critical disorders to ED in CKD. In recent years, clinical practice and scientific research in the field of ED aimed to evaluate whether various circulating markers might have a clinical utility as diagnostic tests or prognostic markers for ED in the CKD population.

Firstly, we will define the basic but fundamental statistic measures in the analysis of diagnostic accuracy. Specificity and sensitivity are statistical measures to evaluate the diagnostic performance of a new, alternative test as compared to the gold standard. In this scenario, true positives are the subjects with a correct diagnosis of the disease (test: positive, disease: positive); true negatives are those that were correctly diagnosed as disease-free (test: positive, disease: positive). False positive indicates subjects not having the disease that were incorrectly diagnosed (test: positive, disease: negative); false negative denotes diseased subjects where the test fails to diagnose them correctly (test: negative, disease: positive). Sensitivity, or the true positive rate, measures the ability of a test to identify true positives (i.e., the performance of a test to classify subjects that actually have the disease) and is calculated by dividing the true positives by the true positives and false negatives. On the other hand, specificity, or true negative rate, measures the ability of a test to identify true negatives (i.e., the performance of a test to classify subjects that are actually disease-free), and is calculated by dividing the true negatives by the true negatives and false positives. Therefore, sensitivity determines the proportion of diseased subjects who will be tested to be positive, and specificity determines the proportion of disease-free subjects who will be tested to be negative. However, a common question in everyday clinical practice is whether a certain subject will have the disease based on the result of the test. This question can be answered using two other statistical measures: the positive predictive value (i.e., the proportion of positive results that are truly positives) and the negative predictive value (i.e., the proportion of negative results that are truly negatives).

A fundamental ability of a diagnostic marker/test is the discriminatory power or accuracy; that is, the ability of this marker to correctly discriminate/categorize subjects as healthy and diseased. This classification demands dichotomous diagnostic tests, which give a “yes or no” answer to the clinical question of presence or absence of the disease. However, the diagnostic markers/tests are usually quantitative that are expressed in continuous terms and need to be categorized. A receiver operating characteristic (ROC) curve analysis evaluates the discriminatory power (or diagnostic accuracy) of a quantitative test/marker in the range of all its values, when the disease status is correctly defined using a gold, reference method. Ideally, the performance (i.e., the accuracy) of a ROC curve as derived from a model (based on one or more diagnostic factors) in a given training set of patients, needs to be formally validated in an independent (external) set of patients with similar characteristics. By definition, the training set cannot be used to evaluate overfitting/underfitting. Therefore, the formal comparison of two ROC curves obtained in the training and in the validation set can be useful to this scope. The magnitude of the difference between the training and validation metrics is an indicator of overfitting when the gap is large and positive (i.e., the ROC curve in the training set is higher than that in the validation set) and underfitting when the gap is large and negative (i.e., the ROC curve in the training set is lower than that in the validation set). Moreover, this analysis allows the identification of an optimal cut-off value of the diagnostic test/marker that will maximize the sensitivity and specificity of the test [3]. The value and utility of the ROC analysis include the comparison of the accuracy between two or more biomarkers for the same disease at the same time and the evaluation of the accuracy of multivariate risk scores. Two ROC curves, as derived from a different set of biomarkers in the same population, can be compared between them with an appropriate statistical test [4] available in several types of statistical software.

Besides the diagnosis, a ROC analysis is also useful for prognosis assessment and might estimate the predictive value of a biomarker for a certain event/disease [5,6]. The ROC analysis was first used from radar operators in World War II to help them decide whether a blip on their radar corresponded to a noise or an actual object, and it was later applied to diagnostic research. The use of ROC curves’ analysis in the scientific literature has gained exponential growth during the past two decades. During 1990–2000, about 1000 papers were published in Medline using a ROC analysis; this number was five times increased in the next decade and during the last decade (2011–2021) nearly 2800 papers regarding ROC curves were annually published. The same trend applies to the area of ED: searching Medline, using the search terms “Endothelial dysfunction” AND “Marker” AND “CKD or ESKD or Hemodialysis” in the title or abstract generated 172 results. Since ED, when established, is very difficult to be managed, the ideal biomarker should be accurate and predict the onset of ED early and with high accuracy. Having this in mind, numerous biomarkers of ED have been proposed and studied. Therefore, it is quite common in this field of interest to evaluate the accuracy or predictive ability of a certain marker, as a potential test to diagnose a certain clinical endpoint. In these settings, the ROC analysis is a key tool in the evaluation of the discriminatory power of ED markers [7].

In this paper, through the presentation and interpretation of a series of practical examples in ED research, we address the principles, fundamentals, advantages and limitations regarding the interpretation of the ROC analysis in clinical studies involving CKD and ESKD patients. Particularly, we will demonstrate the use of this analysis to assess the discriminatory power of diagnostic tests and the predictive value of biomarkers, compare their accuracy, determine optimal cut-off points for these tests and evaluate the accuracy of multivariate risk scores.

## 2. Examples

### 2.1. Example 1

In CKD, OS occurs early, progresses along with the deterioration of kidney function and is thought to promote ED and vascular calcification (VC), through oxidative modification of low-density lipoprotein (LDL) to oxidized LDL (oxLDL) within the vessel wall. The oxidation of LDL is the first, crucial step towards ED and atherosclerosis. However, the data regarding the association between oxLDL and VC in CKD patients remain limited. To evaluate whether oxLDL is an accurate biomarker for the diagnosis of VC in uremia, in a hypothetical cross-sectional clinical study, we enrolled a cohort of 120 predialysis CKD subjects (stages 2–5) and obtained measurements of circulating oxLDL for each patient. We aimed to estimate the classification accuracy by a ROC analysis as we had to determine the disease status for all subjects and classify our cohort into two groups, the diseased (those with VC) and the non-diseased group (those with no VC), by a gold standard method. To ensure that the classification was correct, we obtained autopsy data from the abdominal aorta of our patients, and VC was determined as the presence or absence of calcification within the artery. Since the definition of VC was based on an autopsy method, there was no overlapping between CKD patients with and without VC, thus indicating that aortic autopsy perfectly discriminated the diseased from the non-diseased (true positive rate = 100%, true negative rate = 100%, accuracy = 100%). To evaluate the discriminatory ability of a quantitative test (such as oxLDL for assessing VC) across a series of its values and determine the optimal threshold, we constructed a ROC curve. In this hypothetical cross-sectional study, 120 CKD patients were recruited to evaluate the overall accuracy of oxLDL to discriminate patients with and without VC as assessed by aortic autopsies. Hypothetically, the best cut-off value of oxLDL to identify patients with VC was 70 U/L. The diagnostic vale of the oxLDL threshold of 70 U/L to identify VC in CKD patients is presented in Table 1.

Based on Table 1, we can calculate the diagnostic value indices of oxLDL, as follows:

Sensitivity: 62/80 = 0.775 = 77.5%

Specificity: 32/40 = 0.80 = 80%

False positives (1-specificity):100 − 80 = 20%

Positive predictive value: 62/70 = 0.886 = 88.6%

Negative predictive value: 32/50 = 0.64 = 64%

Accuracy: 62 + 32/120 = 0.783 = 78.3%

The ROC curve is a graphical plot that combines pairs of sensitivity (true positive rate) on the *y* axis and the complement of specificity (1—specificity, false positive rate) in the *x* axis, corresponding to several of the cut-off values covering the complete range of possible values that this test/marker might take. Since both specificity and sensitivity are not affected by the proportion of the diseased subjects, the ROC curve analysis is also unaffected by the prevalence of the disease. 

**Figure 1:**
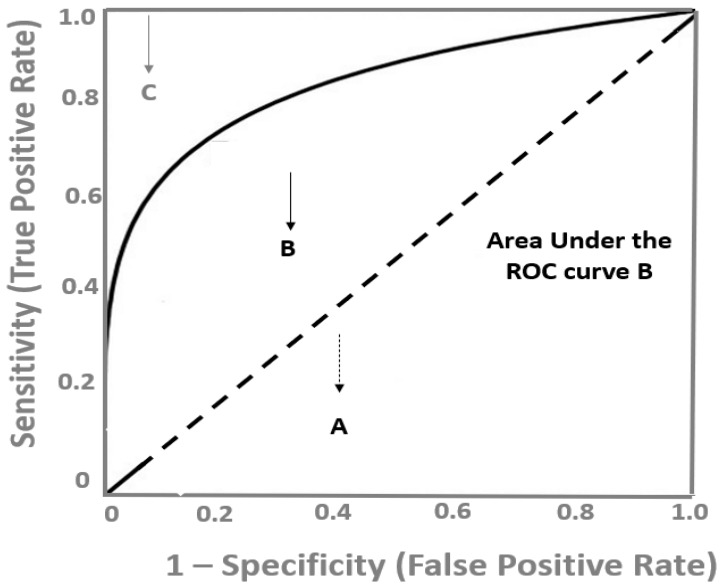
Receiver operating characteristic (ROC) curves showing A: a diagnostic test with the lowest discriminatory ability, which is no better than chance, area under the curve (AUC) = 0.5, B: a test with a modest discriminatory ability and C: a perfect, accurate diagnostic test (highest sensitivity and specificity), AUC = 1.0.

As depicted in Figure 1, a diagnostic test with a high discrimination ability for identifying the diseased subjects has a ROC curve close to the upper left corner of the graph, whereas the closer the ROC plot is to the diagonal reference line (also named as the chance line), the lower the diagnostic accuracy of the test. The overall discriminatory power of a test is assessed by the area under the ROC curve (AUC), which is widely recognized as a global estimate of diagnostic accuracy [8]. In Figure 1, the AUC of the B test is depicted as the grey area under this curve. The maximum value the AUC might take is 1.0, indicating a theoretical, ideal test with 100% specificity and 100% sensitivity (perfect discrimination) and the minimum value is 0.5, which corresponds to no discriminative power at all (50% specificity and 50% sensitivity), which is represented by the area under the diagonal, reference line (Figure 1). There are various proposed interpretations for AUC values, but generally, ROC curves with an AUC above 0.75 might be considered as clinically useful and above 0.85 as having strong diagnostic accuracy, and therefore, potential clinical utility [7].

The choice of the ideal cut-off value in a ROC curves analysis should ensure the highest sensitivity and specificity. However, it is usually a trade-off between specificity and sensitivity and the choice of a higher sensitivity is at the cost of lower specificity. Therefore, the best cut-off value is determined by clinical and probabilistic considerations. To determine the optimal cut-off point of ox-LDL to distinguish CKD patients with VC from those without VC, we determined the sensitivity and specificity over a range of different cut-off points (Table 2).

**Figure 2:**
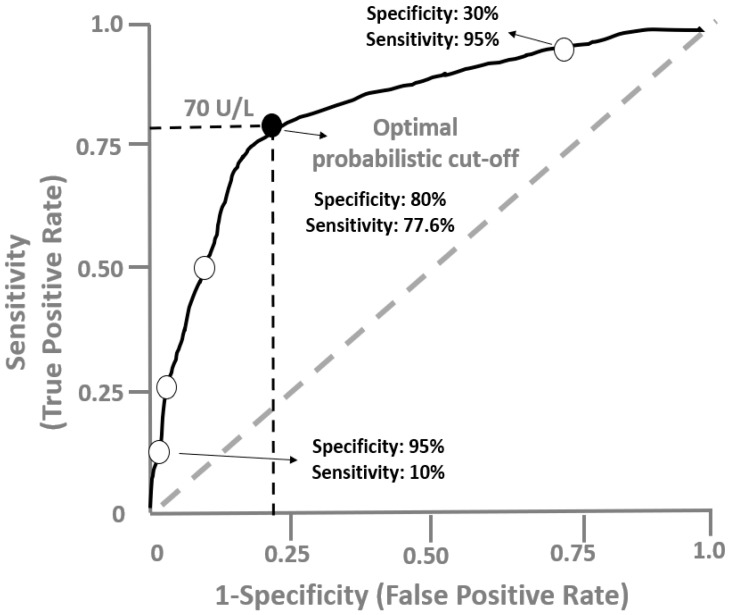
Receiver operating characteristic curves showing the performance of oxLDL for the diagnosis of vascular calcification in CKD patients, in the hypothetical example 1. The white circles over the curve correspond to a series of oxLDL values used to construct the curve and the black circle represents the best probabilistic cut-off value of oxLDL (70 U/L).

Based on Figure 2, which shows the ROC curve for oxLDL in the hypothetical cross-sectional study, a value of 70 U/L might be considered an optimal cut-off point to distinguish CKD patients with VC from those without VC. From a probabilistic point of view, we can use the co-ordinates of the ROC curve to determine the best oxLDL cut-off that provides the maximum discrimination or else the maximum sensitivity and specificity. In this example, this corresponds to the value of 70 U/L, which is the optimal probabilistic cut-off value and provides 80% specificity and 77.5% sensitivity. This could be interpreted as follows: among CKD patients, those with circulating oxLDL above 70 U/L probably have VC and those with oxLDL below 70 U/L more likely do not have VC. However, from a clinician point of view, it might be requested to minimize the false positive rate, and thus, set the specificity at 95% (only 5% will be diagnosed as false positives; that is, as diseased, although not having VC). In the ROC curve (Figure 2), the oxLDL value that provides this specificity corresponds to a low sensitivity of 10%. On the other hand, if a clinician is interested in improving the identification of CKD subjects with VC at the maximum, an oxLDL cut-off value with high sensitivity is needed. If we set the sensitivity at 95%, the oxLDL value that provides this sensitivity corresponds to a modest specificity of 30% (Figure 2). It should be noted that the ROC performance and the optimal cut-off value of a given diagnostic test might change when the test is applied to different patient populations; for example, in non-CKD or ESKD populations, the cut-off value of 70 U/L and the diagnostic indices of oxLDL for identifying VC cannot be applied.

### 2.2. Example 2

Since subclinical overhydration is associated with increased OS, hypertension and CV events in ESKD patients, it is crucial to be recognized early and managed in these patients. Alexiadis et al. [9] aimed to investigate and compare the accuracy of several available techniques for evaluating the hydration status in 53 ESKD patients undergoing maintenance hemodialysis (HD), thrice weekly. In all patients, before and after a HD session, the hydration status was assessed by four different techniques: indexed inferior vena cava diameter (IVCDi), continuous blood volume monitoring (Crit-line), bioelectrical impedance analysis (BIA) and lung comets score with lung ultrasonography. In this setting, to determine the “true disease status”—that is the overhydrated patients—the authors chose IVCDi as the gold standard reference technique and all patients were categorized to overhydrated or underhydrated, based on the results of this test. When determining the gold standard, two major pitfalls should be avoided: first, the measurement error that occurs when there is no true gold standard or when the one used might be flawed. Second, verification bias, which might occur when the gold standard test is accurate only when evaluated in subjects with a known disease status. In the study by Alexiadis et al. patients were characterized as overhydrated when IVCMi was above 11.5 mm/m^2^ and patients were classified accordingly, and based on the results of Crit-Line, BIA and lung comets score, three different thresholds were set for each test. The ROC analysis, besides evaluating the discriminatory ability of a certain test, can also compare more than two methods at once, using a reference method.

**Figure 3:**
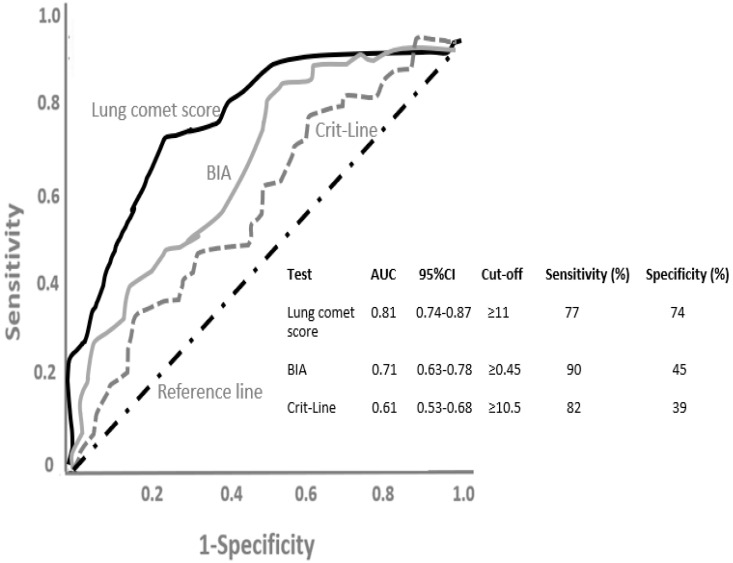
Receiver operating characteristic (ROC) curves evaluating the performance of lung comet score, bioelectrical impedance analysis (BIA) and continuous blood volume monitoring (Crit-Line) test in predicting overhydration, as determined using the indexed inferior vena cava diameter (IVCDi) as the reference method, in hemodialysis patients, as described in the study by Alexiadis et al. in example 2. AUC, area under the curve; CI, confidence interval.

Figure 3 shows the ROC curves evaluating the performance of lung comet score, BIA and Crit-Line test in predicting overhydration, as determined using IVCDi as the reference method, in HD patients. The lung comet score showed more promising results predicting overhydration, as assessed by the AUCs (0.81 for lung comet score, 0.71 for BIA and 0.61 for Crit-Line). Moreover, the difference between AUCs was statistically significant (lung comet score vs. BIA: 0.100, *p* = 0.032), (lung comet score vs. Crit-Line: 0.20, *p* = 0.001). Since 0.50 is the lowest AUC value, a given test has discriminatory power if the 95% confidence interval (CI) of the AUC does not include this value. In Figure 3, the 95%CI for the lung comet score was 0.74–0.87, for BIA 0.63–0.78 and for Crit-Line 0.53–0.68. Of note, the ROC curve for Crit-Line was very close to the diagonal reference line and the AUC was very close to 0.5, indicating that this test had very low diagnostic power. However, the AUC for the lung comet score was 0.81, indicating that if we theoretically selected random pairs of overhydrated and not overhydrated HD patients, the test result (lung comet score) would be higher 81% of the times in the overhydrated patients. In Figure 3, it also shown that at the optimal threshold of ≥11, the lung comet score displayed a sensitivity of 77% and a specificity of 74%, whereas BIA at the cut-off of ≥0.45 presented a sensitivity of 90% and a specificity of 45% and Crit-Line at the cut-off of ≥10.5 presented a sensitivity of 82% and a specificity of 39%.

### 2.3. Example 3

A logistic regression analysis might be used to describe the association between an independent variable and a dependent one. In multiple regression models, we can calculate the regression coefficients, statistical significance and the 95%CIs of independent variables (that can be continuous or not) for predicting the dependent variable; that is, a dichotomous variable that can only take the values of 0 = non-diseased or 1 = diseased. In a recent study [10], aiming to examine the possible association between biomarkers of OS and inflammation and cardiac status, 100 patients presenting with non-ST-elevation myocardial infraction (NSTEMI) at the emergency department were enrolled. Ejection fraction (EF), troponin, interleukin 6 and 10 (IL-6, IL-10), myeloperoxidase (MPO), high-sensitive C-reactive protein (CRP) and fibrinogen were assessed at admission, along with other markers of cardiac (alanine transaminase, aspartate transaminase, brain natriuretic peptide, creatinine phosphokinase, creatinine phosphokinase myocardial band) and kidney function (eGFR and albuminuria). A multiple logistic regression analysis was used to determine independent predictors (independent variables) of patients with the worst cardiac status after the NSTEMI, determined as those with high troponin (dependent variable) and low EF (dependent variable) with two separate models. MPO, IL-10 and AST were independent predictors of high troponin (β = −1.69, 95% CI −3.1 to −0.24, *p* = 0.02, β = 0.15, 95% CI 0.04–0.25, *p* = 0.006, β = 0.04, 95% CI 0.02–0.06, *p* = 0.001, respectively), whereas Hs-CRP, BNP and eGFR were independent predictors of low EF (β = −0.11, 95% CI −0.18 to −0.05, *p* = 0.001, β = −0.011, 95% CI −0.018 to −0.003, *p* = 0.004, β = 0.12, 95% CI 0.012–0.22, *p* = 0.029, respectively).

**Figure 4:**
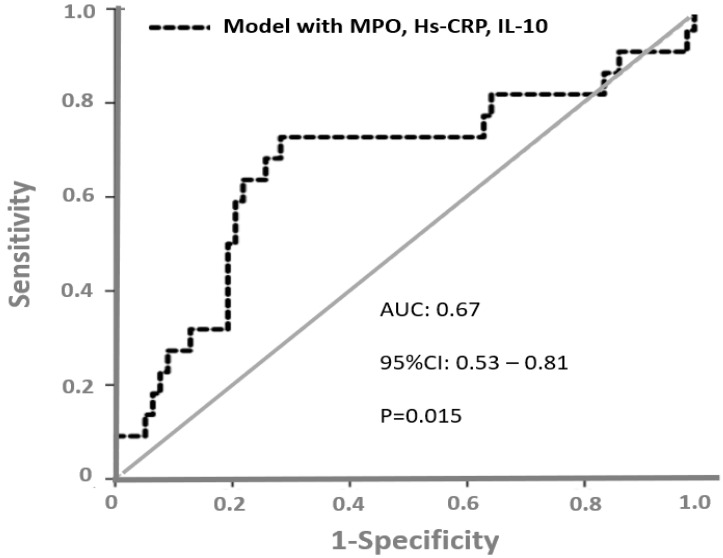
ROC curves showing the discriminatory performance of a risk predictive model including MPO, IL-10 and hs-CRP to discriminate patients with the worst cardiac outcome after NSTEMI, in example 4, taken from the study by Roumeliotis et al.

The authors found that MPO and IL-10 were independent predictors of high troponin and Hs-CRP predicted low EF. To determine patients with the worst cardiac outcome (assessed by low EF and high troponin), the authors constructed a predictive risk model including MPO, IL-10 and Hs-CRP. A ROC curve analysis (Figure 4) showed that the AUC of this risk predictive model to discriminate patients with the worst cardiac outcome was 0.67 (95%CI = 0.53–0.81, *p* = 0.015).

In another paper [11], the authors investigated the relationship between cardiac natriuretic peptides (atrial natriuretic peptide [ANP] and brain natriuretic peptide [BNP]) and norepinephrine (NE) with LAV changes over time in 199 dialysis patients. The ROC curve area for predicting the left atrial volume (LAV) changes (>3 mL/per year) of a model based on standard risk factors was 0.72. Plasma BNP (+12%; *p* = 0.004), ANP (+8%; *p* = 0.03), NE (+8%; *p* = 0.05) and midwall fraction shortening (+8%; *p* = 0.05) increased the area under the ROC curve to a significant extent. The authors concluded that BNP and ANP predict LAV changes over time in dialysis patients and that the measurement of the plasma concentration of these compounds might be useful for guiding treatment in this patient population.

## 3. Conclusions

The ROC curve analysis is a statistical method to assess the diagnostic ability of a test or marker to discriminate between subjects who present a given disease and those who do not. This analysis identifies the optimal cut-off value of a marker, allows the direct comparison of the diagnostic accuracy of two or more markers at the same time and can be also used for prognostic purposes. Scientific research in the area of oxidative medicine commonly focuses on the identification of the clinical utility of biomarkers of OS. Under this perspective, a ROC analysis is an essential statistical tool in oxidative medicine and its use has gained exponential growth during the past decade.

## Figures and Tables

**Table 1:** Diagnostic vale of the oxLDL threshold of 70 U/L to identify VC in CKD patients, in the hypothetical example 1.

Test Result	VC Present	VC Absent	Total
Positive: oxLDL ≥ 70 U/L	62	8	70
Negative: oxLDL < 70 U/L	18	32	50
	80	40	120

**Table 2:** Calculated sensitivity, specificity for determining the optimal cut-off point for oxLDL, in order to diagnose VC in CKD patients.

Cut-Off Points of oxLDL (U/L)	Sensitivity (%)	Specificity (%)
30	10	95
50	25	93
60	50	85
70	77.6	80
80	95	30

## Data Availability

Not applicable.
